# Extraperitoneal Open Radical Cystectomy: A New Standard in Frail Patients with Muscle-Invasive Bladder Cancer?

**DOI:** 10.3390/clinpract14060201

**Published:** 2024-11-24

**Authors:** Daniel Porav-Hodade, Silvestru-Alexandru Big, Vlad-Ilie Barbos, Bogdan Gherle, Ernő Jerzicska, Victor Ona, Bogdan-Ovidiu Feciche

**Affiliations:** 1 Department of Urology, George Emil Palade University of Medicine, Pharmacy, Science, and Technology of Târgu Mureș, 540139 Târgu Mureș, Romania; daniel.porav-hodade@umfst.ro; 2 Department of Urology, Clinical County Hospital Mures, 540136 Târgu Mures, Romania; 3 Department of Urology, Bihor Emergency Clinical County Hospital, 410169 Oradea, Romania; vlad.barbos@umft.ro (V.-I.B.); feciche.bogdanovidiu@didactic.uoradea.ro (B.-O.F.); 4 Doctoral School of Biological and Biomedical Sciences, University of Oradea, 1 University Street, 410081 Oradea, Romania; gherlebogdandaniel@gmail.com; 5 Department of Cardiovascular Surgery, Bihor Emergency Clinical County Hospital, 410169 Oradea, Romania; spitalul.judetean@rdsor.ro; 6 Department of Urology, Clinical Municipal Hospital, 400139 Cluj-Napoca, Romania; contact@uropoint.ro; 7 Department of Urology, Faculty of Medicine and Pharmacy, University of Oradea, 1 University Street, 410087 Oradea, Romania

**Keywords:** bladder cancer, extraperitoneal open radical cystectomy, reperitonization, frailty

## Abstract

**Background/Objectives**: Radical cystectomy (RC) represents one of the most complex and morbid surgical procedures in the field of Urology. Extraperitoneal open RC has emerged as an alternative to the traditional transperitoneal approach for the treatment of muscle-invasive bladder cancer. Frailty is one of the most important risk factors for perioperative morbidity and mortality, and this category of patients can benefit the most from the extraperitoneal approach. The purpose of this study was to evaluate the feasibility and the safety of extraperitoneal open RC in our experience; **Methods**: We retrospectively collected the data of 75 frail patients who underwent an extraperitoneal open RC, performed by a single experienced surgeon. We assessed their frailty status using the simplified frailty index (sFI). We recorded data regarding general characteristics, intraoperative, pathological, and postoperative complications, and mortality (within 90 days); **Results**: We analyzed 61 males and 14 females with an sFI equal to or higher than 3. The median age was 77 years. Fifty-one patients had an ASA score of 3 or more. Sixty procedures were with radical intention, while fifteen were palliative. Cutaneous ureterostomy was performed in 70 cases and extraperitonized ileal conduit in five cases. The median operative time was 150 min. The median blood loss was 400 mL. The median time to flatus was 2 days. The median postoperative stay was 7 days. Thirteen patients had Clavien–Dindo III or IV complications. Two patients died in first 90 days postoperatively; **Conclusions**: The extraperitoneal open RC in frail patients was demonstrated to be a feasible and safe alternative approach in definitive treatment or a palliative setting in our experience.

## 1. Introduction

Bladder cancer (BCa) is one of the most common urological malignancies [1], being the 9th most common cancer worldwide [2]. In BCa, approximately three-quarters of the cases are found in men [3]. In the past decade, the incidence of BCa in Europe has increased, while mortality has decreased. In Asia, however, the opposite has occurred, with a decrease in incidence but an increase in mortality due to BCa in men [4]. Unfortunately, the 5-year survival rate for BCa has remained relatively low compared to other urological cancers [5]. Around 75% of BCa patients present with disease limited to the mucosa (stage Ta, CIS) or submucosa (stage T1) [6]. The remaining patients, unfortunately, present with MIBC or metastases at the time of their initial evaluation.

According to the international guidelines, radical cystectomy (RC) is the recommended treatment for localized muscle-invasive bladder cancer (MIBC), very high-risk NMIBC (non-muscle-invasive bladder cancer), and BCG (Bacillus Calmette–Guérin)-unresponsive NMIBC [7,8,9].

The transperitoneal approach is the standard method for RC, although the gastro-intestinal, wound-related, and infectious complications contribute to the still increased morbidity of the procedure [10].

Extraperitoneal RC was proposed with the aim of reducing morbidity. The peritoneum is not entered in the initial steps of the procedure, but only the serosa covering the bladder dome is ultimately excised, followed by reperitonization. This approach presents several potential advantages compared to the traditional transperitoneal route, including reduced surgical trauma to the abdominal cavity, fewer postoperative complications, and a potentially quicker recovery times. The extraperitoneal approach helps to minimize the risk of ileus, peritonitis, and adhesions, which are especially worrisome in frail patients [10,11].

Although it was initially concerning regarding the radicalness of the surgery with the extraperitoneal approach, there have been comparative studies demonstrating that the two approaches have similar oncological outcomes (surgical margins, local recurrence, distant metastasis, and overall survival.) [12].

More than chronological age, frailty is a distinct entity and refers to an increased vulnerability to stressors due to the cumulative effects of various conditions over time, independent of the normal aging process. It is one of the most important predictors of perioperative morbidity and mortality in RC [13].

The aim of this study was to evaluate the perioperative results and complications of open extraperitoneal radical cystectomy in frail patients with muscle-invasive bladder cancer.

## 2. Materials and Methods

### 2.1. Study Population

We retrospectively included 75 patients considered frail who underwent an open radical cystectomy by the extraperitoneal approach between March 2014 and March 2024. The assessment of frailty was made by calculating the simplified frailty index, as described by Sathianathen et al. [14].

Sixty procedures were elective with curative intent for nonmetastatic MIBC, while in 15 cases, the procedure was performed in a palliative setting (e.g., bladder tumor with refractory hematuria). In all cases, the surgical intervention was performed by the same surgeon, with high experience in open uro-oncological surgery.

The clinical staging was established based on preoperative Computed Tomography of the chest, abdomen, and pelvis.

We recorded the patients’ characteristics in our database during the hospital stay for perioperative data: gender, age, body mass index (BMI), clinical TNM stage, preoperative laboratory test results, ASA and frailty scores, intraoperative data (total operative time, estimated blood loss, number of patients who required a blood transfusion, type of urinary diversion, type of anesthesia), intraoperative incidents, immediate postoperative complications, postoperative laboratory tests, time to first flatus (recovery of bowel function), and length of postoperative hospital stay.

The postoperative complications occurring after discharge (within 90 days after surgery) were assessed during follow-up visits. The postoperative complications were graded according to the Clavien–Dindo classification [15].

All patients provided their informed consent for participation in our study.

### 2.2. Surgical Technique

The preoperative anesthetic risk was assessed using the ASA (American Society of Anesthesiologist) score. Forty-six patients underwent general anesthesia (with orotracheal intubation), while 29 patients underwent epidural anesthesia.

Subjects with a negative urine culture received antimicrobial prophylaxis one hour before skin incision, while those with a preoperative positive urine culture received at least 3–5 days of antibiotics preoperatively according to an antibiogram [16]. Mechanical and pharmacological antithrombotic prophylaxis was perioperatively applied in all cases. Bowel preparation before surgery was not applied at all, except in the five cases where an ileal conduit was performed.

Patients were placed in the Trendelenburg position (15 degrees) with the surgical bed slightly flexed just above iliac crest. The Retzius space was entered using a median infra-umbilical incision. The pelvic parietal peritoneum was bluntly pushed cephalad up to the level of common iliac vessel. The vas deferens was dissected and sectioned to allow a better medial mobilization of the peritoneum. The avascular lateral perivesical space on each side was bluntly developed up to the endopelvic fascia, as well as the retrocolic space. The ureters were isolated on vascular loops where they crossed the iliac vessels and their dissection was proceeded with as caudally as possible. The superior vesical artery was identified, clipped or ligated, then sectioned. The retroureteral bladder pedicles, that allow an extensive posterolateral mobilization of the bladder, were isolated, ligated, and sectioned. The endopelvic fascia was incised on both sides, then the lateral prostatic pedicles were isolated, ligated, and sectioned. Then, the dissection of prostate apex proceeded. The puboprostatic ligaments were sectioned, and the dorsal vascular complex Santorini and the urethra were ligated and sectioned.

The posterior side of the prostate was dissected off the Denonvillier fascia, then the entire specimen could be detached off the inferior peritoneum. Only that portion of peritoneum that covers the posterior bladder surface was excised en bloc with the specimen.

With the exception of emergency procedures performed with hemostatic purpose only, standard lymph node dissection (LND) proceeded after completion of radical cystectomy.

The ureterostomy was performed, then the continuity of peritoneum was reestablished using absorbable sutures before the abdominal wall was closed.

In cases where ileal conduit was performed, the peritoneum was closed around the mesentery of the conduit.

### 2.3. Statistical Analysis

The median and range for continuous variables (as they were not normally distributed according to the Shapiro–Wilk test) and frequencies and percentages for categorical variables were used. For the statistical studies, IBM SPSS 19 and Microsoft Excel 2019 were utilized.

This study was conducted in accordance with the Ethics Committee of the Bihor Emergency Clinical County Hospital, 410169 Oradea, Romania code 27833, Approval Date: 12 September 2024.

## 3. Results

The general characteristics of patients are presented in Table 1. A total of 75 patients, 61 males and 14 females, that met the frailty criteria (sFI equal or higher than 3) were analyzed. The median age was 77 years (range: 59–84 years).

Twenty patients had a degree of obstructive renal failure (serum creatinine over 2 mg/dL and hydronephrosis). Thirty-five patients had preoperative moderate or severe anemia (Hb < 10 g/dL) and thirty-three had hypoalbuminemia. The median BMI was 26 kg/m^2^ (range: 17–35 kg/m^2^).

Regarding the preoperative anesthesia risk, 24 patients had an ASA score of 2 and 51 had an ASA score of 3 or higher.

The surgical procedure had radical intention in 60 cases and a palliative purpose (e.g., for refractory hematuria) in 15 cases, when standard lymph node dissection was not performed. Bilateral direct ureterostomy was performed in 65 cases, while extraperitonized ileal conduit was used for urinary diversion in ten cases. Forty-six patients underwent only regional anesthesia (epidural or combined spinal/epidural anesthesia) and 29 patients underwent general anesthesia.

The intraoperative and postoperative results are shown in Table 2. The median operative time was 150 min (range: 90–210 min). The median blood loss was 400 mL (range: 200–1500 mL). Thirty percent of patients needed blood transfusions. The median time to flatus (postoperative ileus) was 2 days (range: 1–7 days). The median postoperative hospital stay was 7 days (5–14 days). Thirteen patients had Clavien–Dindo III or IV complications. Two of them required surgical reintervention where intestinal lesions related to the reperitonization were identified and solved. A lymphocele was encountered in six cases; five of them were drained percutaneously and one was fenestrated laparoscopically. Nine cases were complicated by superficial (suprafascial) wound dehiscence, treated with daily wet-to-dry dressings, antibiotics, and eventually secondary suturing in three cases.

Four patients had ileus (postoperative nausea/vomiting and abdominal distension, necessitating nasogastric tube insertion, discontinuation of oral intake, and/or parenteral nutrition). Postoperative venous thromboembolism was encountered in two cases, while one patient presented with acute coronary syndrome.

Rectal injury was encountered in one case, that was intraoperatively recognized and repaired by a primary suture.

Two patients died in first 30 days postoperatively, but there were no intraoperative deaths.

The pathological results are summarized in Table 3.

## 4. Discussion

The ageing of the population is making the management of MIBC in older individuals a challenging issue. Bladder cancer (BCa) is more likely to affect this category of patients, as the age-specific incidence rates gradually increase after 50 years of age [17].

RC is the gold-standard treatment of MIBC, with alternatives like transurethral resection alone or trimodality treatment [18] having similar oncological outcomes in selected cases only [7].

The complication rates after standard transperitoneal RC remain high, especially in co-morbid patients. In a study on the early morbidity of radical cystectomy, Shabsigh et al. reported a Clavien–Dindo III–V complication rate of 13%. Among these, the most frequent complications were gastrointestinal (29%), infectious (25%), and wound-related (15%) complications [19].

Even though conventional transperitoneal radical cystectomy is the standard approach for muscle-invasive bladder cancer [20], the procedure is associated with significant morbidities such as urinary leakage, ileus, and infections [21]. The cause is supposedly the deperitonized pelvic wall that comes in contact with the bowel serosa, combined with the increased exposure of the bowels to the atmosphere, resulting in an increased local inflammatory reaction and, in consequence, reduced bowel peristalsis, increased ileus duration, obstruction, distention, and increased pain [21,22].

An extraperitoneal retrograde approach for RC and pelvic LND was published for the first time in 1999 by Kulkarni et al. [23] to reestablish the natural compartmentalization between the gastrointestinal and the urinary tracts and to minimize the handling and prolonged exposure of the intestines to the air. Although there are several differences between our technique and the original one, the main principle regarding peritoneal continuity was preserved. Only the peritoneum of the bladder dome surface was excised en bloc with the specimen, then the remaining serosa was completely closed (if the cutaneous ureterostomy was performed) or closed around the mesentery of the conduit. The extraperitoneal RC has been adopted by many surgeons, with various modifications [6].

In the retrospective study of Kulkarni et al. published in 2018, they compared the extraperitoneal approach with the transperitoneal approach in open RC. They found a statistically significantly lower rate of ileus (5% vs. 15.8%, *p* < 0.001) and a delayed intestinal obstruction rate (1.7% vs. 7.8%, *p* = 0.002) in the first group. The rates of other early and delayed complications were slightly lower after extraperitoneal RC, but the results were not statistically significant [21].

Another study published by Özkaptan et al. in 2020 [24] that compared the perioperative outcomes of extraperitoneal antegrade and transperitoneal open RC with ileal conduit performed in their center. They found statistically significantly superior results regarding the postoperative hospital stays (median: 9 versus 10 days, *p* = 0.042), bowel function recovery (median: 3 versus 4 days, *p* < 0.01), and the rate of postoperative ileus (5.8 versus 19.2%, *p* = 0.043) in the first group.

A systematic review and meta-analysis compared the perioperative outcomes after transperitoneal versus extraperitoneal RC in the treatment of BC, including 1207 patients (619 in the first group and 588 in the other), was published in 2023 by Leonardo et al. [20]. The patients undergoing extraperitoneal RC had a significantly lower occurrence of postoperative ileus, but the results regarding the rate of the Clavien–Dindo III–V complications, estimated blood loss, operative time, postoperative hospital stay duration, and infections were not conclusive, without statistically significant differences between the groups.

The urinary diversion chosen for the most patients was cutaneous ureterostomy, which can partially explain the lower rates of gastrointestinal complications encountered in our study group when compared with the literature data regarding this type of approach [25,26].

The role of readaptation of the posterolateral peritoneum after RC with LND was evaluated in a prospective randomized trial that included 200 patients. The patients undergoing this technique had less postoperative pain, earlier recovery of bowel function, and fewer complications in the first 30 days postoperatively [27].

The management of MIBC in older and frail patients remains a controversial topic in the literature. Subjects with BCa had, at the time of diagnosis, the highest median age among all cancer types and more chronic comorbidities when compared with the general population (eight versus four comorbidities) [28]. Frailty is an independent entity characterized by an accelerated decline in physical and cognitive resilience to stressors like infections or surgery. It is a strong predictor for perioperative complications, impaired quality of life, and mortality [13].

Radical cystectomy (RC) carries a significant perioperative mortality rate [19], ranging from 0.8% to 3% [29,30]. However, this mortality rate is inversely related to the experience of the surgical centers, with a lower mortality observed in high-volume academic institutions [31].

Given the increased morbidity of the procedure, RC is not widely accepted as a standard treatment for MIBC in these categories of patients. Thus, it is usually considered when the other alternatives are not feasible or have failed. If the perioperative risk is acceptable, many patients with BC are willing to undergo radical surgery. There is no objective stratification of the perioperative risk for major surgery, which contributes to the reluctance of urologists to offer RC in this category of patients [28].

We used the sFI to measure frailty. It has five items, with one point attributed for each of them: functional status, DM (diabetes mellitus), COPD (chronic obstructive pulmonary disease), CCF (congestive cardiac failure), and hypertension that requires treatment. Patients with an sFI more than or equal to 3 have higher rates of Clavien–Dindo III–V complications and early postoperative mortality. There are studies that demonstrate the superiority and the simplicity of this score in predicting the postoperative complications following RC when compared with traditional methods like the ASA score. The score helps us not to necessarily contraindicate the radical surgery, but to select the category of patients that could potentially benefit the most from less morbid approaches and from health-optimizing interventions after surgery [14,32].

Despite initial concerns about the radical nature of surgery using the extraperitoneal approach, comparative studies have shown no statistically significant differences in oncological outcomes between extraperitoneal and transperitoneal radical cystectomy (RC) [12]. The pathological results in our series are comparable with those found in the literature.

The main drawbacks of this study are represented by the retrospective nature of the data collection, the relatively low patient numbers, and the lack of a control group to compare the outcomes.

Although we are in the era of robotic-assisted surgery, we consider that the extraperitoneal open approach can still find a place for frail patients who cannot tolerate general anesthesia, hypercapnia, and/or a steep Trendelenburg position.

## 5. Conclusions

Extraperitoneal open RC in frail patients was demonstrated to be a feasible and safe alternative in definitive treatment or a palliative setting in our experience, with acceptable outcomes. Further prospective, comparative, multicentric studies are needed to generalize the conclusion.

## Figures and Tables

**Table 1:** The general characteristics of patients.

Variable	Value
Age at surgery (years), median (range)	77 (59–84)
Males, *n* (%)	61 (81.3)
Females, *n* (%)	14 (18.7)
BMI [kg/m^2^], median (range)	26 (17–35)
Obstructive renal failure, *n* (%)	20 (26.7)
Moderate–severe anemia, *n* (%)	35 (46.7)
Hypoalbuminemia, *n* (%)	33 (44.0)
ASA score	
ASA 2, *n* (%)	24
ASA 3, *n* (%)	47
ASA 4 *n* (%)	4
Preoperative clinical T stage	
Ta-T1, *n* (%)	2 (2.7)
T2, *n* (%)	48 (64.0)
T3, *n* (%)	21 (28.0)
T4, *n* (%)	4 (5.3)
Preoperative clinical N stage	
N0, *n* (%)	54 (72.0)
N+, *n* (%)	21 (28.0)
Preoperative clinical M stage	
M0, *n* (%)	59 (78.7)
M1a, *n* (%)	7 (9.3)
M1b, *n* (%)	9 (12.0)

BMI = body mass index; ASA = American Society of Anesthesiologists.

**Table 2:** Intraoperative and postoperative results.

Variable	Value
Median operative time (minutes), median (range)	150 (90–210)
Median blood loss (mL), median (range)	400 (200–1500)
Median time to flatus (days), median (range)	2 (1–7)
Blood transfusion, *n* (%)	23 (30)
Urinary diversion	
Cutaneous ureterostomy, *n* (%)	65 (86.7)
Ileal conduit, *n* (%)	10 (13.3)
Intraoperative incidents	
Rectal injury, *n* (%)	1 (1.3)
External iliac vein injury, *n* (%)	1 (1.3)
Obturatory nerve injury, *n* (%)	1 (1.3)
Postoperative complication	
Clavien–Dindo III, *n* (%)	11 (14.7)
Clavien–Dindo IV, *n* (%)	2 (2.7)
Clavien–Dindo V, *n* (%)	2 (2.7)
Duration of postoperative hospital stay (days), median (range)	7 (5–14)

**Table 3:** The general characteristics of patients.

Variable	Value
Pathological T stage	
pT2, *n* (%)	48 (64)
pT3, *n* (%)	23 (30.7)
pT4, *n* (%)	4 (5.3)
Pathological N stage	
Lymph node dissection, *n* (%)	60 (80)
pN0, *n* (%)	45 (60)
pN1, *n* (%)	12 (18)
pN2, *n* (%)	3 (4.4)
Concomitant prostate adenocarcinoma	15 (20.0)
Positive surgical margins, *n* (%)	1 (1.3)

## Data Availability

The data presented in this study are available upon request from the corresponding author. The data are not publicly available due to restrictions.

## References

[1] Sung H., Ferlay J., Siegel R.L., Laversanne M., Soerjomataram I., Jemal A., Bray F. (2021). Global Cancer Statistics 2020: GLOBOCAN Estimates of Incidence and Mortality Worldwide for 36 Cancers in 185 Countries. CA Cancer J. Clin..

[2] Mártha O., Balan D., Porav-Hodade D., Drágus E., Vartolomei M.D., Chibelean C.B., Borda A., Pytel Á., Vida O.Á. (2020). The role of neutrophil to lymphocyte ratio in patients with pTa non-muscle invasive bladder cancer. Rev. Romana De Med. De Lab..

[3] Antoni S., Ferlay J., Soerjomataram I., Znaor A., Jemal A., Bray F. (2017). Bladder Cancer Incidence and Mortality: A Global Overview and Recent Trends. Eur. Urol..

[4] Teoh J.Y.-C., Huang J., Ko W.Y.-K., Lok V., Choi P., Ng C.-F., Sengupta S., Mostafid H., Kamat A.M., Black P.C. (2020). Global Trends of Bladder Cancer Incidence and Mortality, and Their Associations with Tobacco Use and Gross Domestic Product Per Capita. Eur. Urol..

[5] Wong M.C.S., Fung F.D.H., Leung C., Cheung W.W.L., Goggins W.B., Ng C.F. (2018). The global epidemiology of bladder cancer: A joinpoint regression analysis of its incidence and mortality trends and projection. Sci. Rep..

[6] Burger M., Catto J.W.F., Dalbagni G., Grossman H.B., Herr H., Karakiewicz P., Kassouf W., Kiemeney L.A., La Vecchia C., Shariat S. (2013). Epidemiology and risk factors of urothelial bladder cancer. Eur. Urol..

[7] Witjes J.A., Bruins H.M., Carrión A., Cathomas R., Compérat E., Efstathiou J.A., Fietkau R., Gakis G., Lorch A., Martini A. (2024). European Association of Urology Guidelines on Muscle-invasive and Metastatic Bladder Cancer: Summary of the 2023 Guidelines. Eur. Urol..

[8] Babjuk M., Burger M., Compérat E.M., Gontero P., Mostafid A.H., Palou J., van Rhijn B.W.G., Roupret M., Shariat S.F., Sylvester R. (2022). European Association of Urology Guidelines on Non-muscle-invasive Bladder Cancer (Ta, T1, and Carcinoma in Situ). Eur. Urol..

[9] Holzbeierlein J., Bixler B.R., Buckley D.I., Chang S.S., Holmes R.S., James A.C., Kirkby E., McKiernan J.M., Schuckman A. (2024). Treatment of Non-Metastatic Muscle-Invasive Bladder Cancer: AUA/ASCO/SUO Guideline (2017; Amended 2020, 2024). J. Urol..

[10] Aminoltejari K., Black P.C. (2020). Radical cystectomy: A review of techniques, developments and controversies. Transl. Androl. Urol..

[11] De Nunzio C., Cicione A., Leonardo F., Rondoni M., Franco G., Cantiani A., Tubaro A., Leonardo C. (2011). Extraperitoneal radical cystectomy and ureterocutaneostomy in octogenarians. Int. Urol. Nephrol..

[12] You C., Li Q., Yang Y., Qing L., Liu S., Wang Y., Dong Z. (2023). Extraperitoneal Versus Intraperitoneal Radical Cystectomy for Bladder Cancer: A Systematic Review and Meta-Analysis. Ann. Surg. Oncol..

[13] Robinson T.N., Eiseman B., Wallace J.I., Church S.D., McFann K.K., Pfister S.M., Sharp T.J., Moss M. (2009). Redefining Geriatric Preoperative Assessment Using Frailty, Disability and Co-Morbidity. Ann. Surg..

[14] Sathianathen N.J., Jarosek S., Lawrentschuk N., Bolton D., Konety B.R. (2019). A Simplified Frailty Index to Predict Outcomes After Radical Cystectomy. Eur. Urol. Focus.

[15] Dindo D., Demartines N., Clavien P.A. (2004). Classification of Surgical Complications: A New Proposal with Evaluation in a Cohort of 6336 Patients and Results of a Survey. Ann. Surg..

[16] Marano L., Carbone L., Poto G.E., Calomino N., Neri A., Piagnerelli R., Fontani A., Verre L., Savelli V., Roviello F. (2022). Antimicrobial Prophylaxis Reduces the Rate of Surgical Site Infection in Upper Gastrointestinal Surgery: A Systematic Review. Antibiotics.

[17] Zhang Y., Rumgay H., Li M., Yu H., Pan H., Ni J. (2023). The global landscape of bladder cancer incidence and mortality in 2020 and projections to 2040. J. Glob. Health.

[18] Premo C., Apolo A.B., Agarwal P.K., Citrin D.E. (2015). Trimodality Therapy in Bladder Cancer. Urol. Clin. N. Am..

[19] Shabsigh A., Korets R., Vora K.C., Brooks C.M., Cronin A.M., Savage C., Raj G., Bochner B.H., Dalbagni G., Herr H.W. (2009). Defining early morbidity of radical cystectomy for patients with bladder cancer using a standardized reporting methodology. Eur. Urol..

[20] Leonardo K., Mirza H., Seno D.H., Purnomo N., Afriansyah A., Siregar M.A.R. (2023). Transperitoneal vs extraperitoneal radical cystectomy: A systematic review and meta-analysis. PLoS ONE.

[21] Kulkarni J.N., Agarwal H. (2018). Transperitoneal vs. extraperitoneal radical cystectomy for bladder cancer: A retrospective study. Int. Braz. J. Urol..

[22] Zhang Y., Zhou H., Tuo Z.T., Wang J., Sun C., Bi L. (2022). Extraperitoneal laparoscopic radical cystectomy with intracorporeal neobladder: A comparison with transperitoneal approach. World J. Surg. Oncol..

[23] Kulkarni J.N., Gulla R.I., Tongaonkar H.B., Kashyapi B.D., Rajyaguru K.B. (1999). Radical Cystoprostatectomy: An Extraperitoneal Retrograde Approach. J. Urol..

[24] Özkaptan O., Çubuk A., Dinçer E., Şahan A., Kafkaslí A., Akça O. (2020). Extraperitoneal Antegrade vs Transperitoneal Open Radical Cystectomy: Single Center Experiences with 200 Cases. Bladder Cancer.

[25] Soleimani M., Moradkhani E., Masoumi N., Gholivandan J. (2021). Extra-Peritoneal versus Trans-Peritoneal Open Radical Cystectomy—Comparison of Two Techniques in Early Post-Operative Complications. Urol. J..

[26] Jentzmik F., Schostak M., Stephan C., Baumunk D., Lingnau A., Weikert S., Lein M., Miller K., Schrader M. (2010). Extraperitoneal radical cystectomy with extraperitonealization of the ileal neobladder: A comparison to the transperitoneal technique. World J. Urol..

[27] Roth B., Birkhäuser F.D., Zehnder P., Burkhard F.C., Thalmann G.N., Studer U.E. (2011). Readaptation of the Peritoneum Following Extended Pelvic Lymphadenectomy and Cystectomy Has a Significant Beneficial Impact on Early Postoperative Recovery and Complications: Results of a Prospective Randomized Trial. Eur. Urol..

[28] van der Vlies E., Los M., Stijns P.E.F., van Hengel M., Blaauw N.M.S., Bos W.J.W., van Dongen E.P.A., van Melick H.H.E., Noordzij P.G. (2020). Preoperative frailty and outcome in patients undergoing radical cystectomy. BJU Int..

[29] Novara G., Catto J.W., Wilson T., Annerstedt M., Chan K., Murphy D.G., Motttrie A., Peabody J.O., Skinner E.C., Wiklund P.N. (2015). Systematic Review and Cumulative Analysis of Perioperative Outcomes and Complications After Robot-assisted Radical Cystectomy. Eur. Urol..

[30] Lauridsen S.V., Tønnesen H., Jensen B.T., Neuner B., Thind P., Thomsen T. (2017). Complications and health-related quality of life after robot-assisted versus open radical cystectomy: A systematic review and meta-analysis of four RCTs. Syst. Rev..

[31] Jerlström T., Gårdmark T., Carringer M., Holmäng S., Liedberg F., Hosseini A., Malmström P.-U., Ljungberg B., Hagberg O., Jahnson S. (2014). Urinary bladder cancer treated with radical cystectomy: Perioperative parameters and early complications prospectively registered in a national population-based database. Scand. J. Urol..

[32] De Nunzio C., Cicione A., Izquierdo L., Lombardo R., Tema G., Lotrecchiano G., Minervini A., Simone G., Cindolo L., D’orta C. (2019). Multicenter Analysis of Postoperative Complications in Octogenarians After Radical Cystectomy and Ureterocutaneostomy: The Role of the Frailty Index. Clin. Genitourin. Cancer.

